# Hypoalbuminemia Is a Strong Predictor of 30-Day All-Cause Mortality in Acutely Admitted Medical Patients: A Prospective, Observational, Cohort Study

**DOI:** 10.1371/journal.pone.0105983

**Published:** 2014-08-22

**Authors:** Marlene Ersgaard Jellinge, Daniel Pilsgaard Henriksen, Peter Hallas, Mikkel Brabrand

**Affiliations:** 1 Department of Anesthesiology, Sydvestjysk Sygehus Esbjerg, Esbjerg, Denmark; 2 Department of Clinical Biochemistry and Pharmacology, University of Southern Denmark, Odense, Denmark; 3 Department of Anesthesiology, Juliane Marie Centret, Rigshospitalet, Copenhagen, Denmark; 4 Department of Emergency Medicine, Sydvestjysk Sygehus Esbjerg, Esbjerg, Denmark; 5 Department of Emergency Medicine, Odense University Hospital, Odense, Denmark; Medical University of Graz, Austria

## Abstract

**Objective:**

Emergency patients with hypoalbuminemia are known to have increased mortality. No previous studies have, however, assessed the predictive value of low albumin on mortality in unselected acutely admitted medical patients. We aimed at assessing the predictive power of hypoalbuminemia on 30-day all-cause mortality in a cohort of acutely admitted medical patients.

**Methods:**

We included all acutely admitted adult medical patients from the medical admission unit at a regional teaching hospital in Denmark. Data on mortality was extracted from the Danish Civil Register to ensure complete follow-up. Patients were divided into three groups according to their plasma albumin levels (0–34, 35–44 and ≥45 g/L) and mortality was identified for each group using Kaplan-Meier survival plot. Discriminatory power (ability to discriminate patients at increased risk of mortality) and calibration (precision of predictions) for hypoalbuminemia was determined.

**Results:**

We included 5,894 patients and albumin was available in 5,451 (92.5%). A total of 332 (5.6%) patients died within 30 days of admission. Median plasma albumin was 40 g/L (IQR 37–43). Crude 30-day mortality in patients with low albumin was 16.3% compared to 4.3% among patients with normal albumin (p<0.0001). Patients with low albumin were older and admitted for a longer period of time than patients with a normal albumin, while patients with high albumin had a lower 30-day mortality, were younger and were admitted for a shorter period. Multivariable logistic regression analyses confirmed the association of hypoalbuminemia with mortality (OR: 1.95 (95% CI: 1.31–2.90)). Discriminatory power was good (AUROC 0.73 (95% CI, 0.70–0.77)) and calibration acceptable.

**Conclusion:**

We found hypoalbuminemia to be associated with 30-day all-cause mortality in acutely admitted medical patients. Used as predictive tool for mortality, plasma albumin had acceptable discriminatory power and good calibration.

## Introduction

Albumin is a acute-phase protein, is synthesized by the liver and has a number of key functions: It is the primary serum binding protein responsible for transport of various substances, e.g. fatty acids and hormones, it has an anti-thrombotic effect and it is essential in the maintenance of normal plasma colloid oncotic pressure. [1] Normally, albumin has a long half-life (15–19 days), but the plasma albumin can fall by 10–15 g/L in 3 to 5 days in critically ill patients [2].

Hypoalbuminemia have previously been associated with increased short-term mortality, length of hospital stay and complications. [1]–[3] A large prospective study on emergency department patients showed, that the short-term mortality of patients with hypoalbuminemia was three times as high compared to patients with normal albumin, even after adjusting for several confounders. [1] This indicates, that albumin could be a useful predictor of poor outcome of emergency patients.

Previous studies have shown hypoalbuminemia to be associated with increased mortality [1], but have not assessed the predictive power on mortality of low albumin levels in an unselected acutely admitted medical population. The aim of the study was to assess the predictive power of hypoalbuminemia in an unselected cohort of medical patients, and to explore a potential association between hypoalbuminemia and 30-day all-cause mortality.

## Methods

We conducted a prospective, observational, cohort study of all patients admitted to a Danish medical admission unit from October 2008 through February 2009 and February through May 2010 to assess the discriminatory power of hypoalbuminemia and to describe 30-day mortality of patients admitted with hypoalbuminemia.

### Study design and setting

The study was a secondary analysis of data from a large study of all acutely medical patients from the medical admission unit at a regional teaching hospital in Denmark. The hospital is a 460-bed teaching hospital in Western Denmark, which serves a population of approximately 220,000, with all subspecialties of internal medicine represented. Patients could either be admitted by their general practitioner, out-of-hours emergency medical clinics, outpatient clinics, emergency department or by ambulance services. The methods for data collection have been reported in detail [4].

### Participants

Eligible patients were all adults (age ≥15 years) admitted at the medical admission unit within the study period.

### Data sources/measurements

Data on the initial blood tests on each patient was electronically extracted from the electronic health records, and vital signs measured at arrival to the medical admission unit and primary complaint were collected by manual chart review.

We were able to link each patient to several national population based registers using the unique Danish personal identification number. [5] We obtained data on length of stay as well prior and current discharge diagnoses from the Danish National Patient Register, a population based administrative register. [6] Data on deaths were obtained from the Danish Civil Registration System ensuring complete follow-up [7].

### Definitions

We divided the patients into three predefined groups according to their plasma albumin levels (0–34, 35–44 and ≥45 g/L). The cutoffs were defined by the hospital-based limits.

Using the discharge diagnoses from the preceding admissions, we calculated the Charlson Comorbidity Score as a marker for comorbid illness. [8] We chose to use the Charlson Comorbidity Score, as this is the most commonly use scale for comorbidity and it defines several of the known risk factors for hypoalbuminemia including liver, rheumatologic and chronic renal disease.

As a marker of disease severity, we calculated the Worthing Physiological Score (WPS) from the vital signs extracted from the electronic health records. WPS uses baseline vital signs to assess the severity of disease, and have been developed for use in a setting similar to ours.

### Analysis

Data will be presented as median (inter-quartile range [IQR]) or proportions (%). Differences between categorical data will be tested using the Chi-square test.

To clarify the ability of hypoalbuminemia to identify patients at increased risk of dying (discriminatory power), we calculated the Area Under the Receiver Operating Characteristic (AUROC) curve, which is a summary measure of sensitivity and specificity at each possible cutoff. The precision (calibration) was assessed using Hosmer-Lemeshow goodness-of-fit test. The calibration assesses if the observed mortality rate matches the expected rate.

We computed a Kaplan-Meier survival plot of the three levels of albumin and compared differences between groups using the log-rank test. We also calculated logistic regression models where the outcome was 30-day all-cause mortality and the exposure albumin level. Model 1 was an unadjusted analysis of the exposures where the reference was normal albumin (35–44 g/l). Model 2 was a multivariable analysis of the exposure adjusting for sex, age, Charlson Comorbidity Score (continuous) and WPS score (continuous).

Unfortunately, we did not have access to albumin levels prior to admission. In an attempt to assess the impact of chronic inflammation on the predictive power of hypoalbuminemia, we performed a logistic regression analysis using C-Reactive Protein (CRP), moderate and severe liver disease, rheumatologic or renal disease, any malignancies and diabetes with complications, all defined according to the Charlson index [8] as covariates.

Stata 13.1 (Stata Corp., Texas, USA) was used for analyses.

### Ethics

The study was approved by the Danish Data Protection Agency. Approval from the Regional Committee on Health Research Ethics was not required by Danish regulations. No written informed consent was given by participants for their clinical records to be used in this study. Patient information was anonymized and de-identified prior to analysis.

## Results

### Participants

A total of 5894 patients were included, 2950 (50.1%) were female and median age was 65 years (49–77). The median length of stay was 2 days (1–6) and 332 (5.6%) patients died within 30 days of admission. The median overall Charlson comorbidity index was 1 (0–3), see table 1.

**Table 1:** Demographic information on patients, n = 5451.

Variable	Overall	Low	Normal	High	Albumin
		(0–34 g/L),	(35–44 g/L),	(>44 g/L),	missing,
	n = 5894	n = 742	n = 3840	n = 869	n = 443
Female, n (%)	2950 (50.1%)	370 (49.9%)	1910 (49.7%)	443 (51.0%)	227 (51.2%)
Age, median years (IQR)	65 (49–77)	74 (64–83)	67 (53–77)	49 (34–64)	60 (41–74)
Length of stay, median days (IQR)	2 (1–6)	6 (2–12)	2 (1–6)	1 (0–2)	1 (0–3)
Charlson comorbidity score, median score (IQR)	1 (0–3)	2 (1–4)	1 (0–3)	0 (0–1)	1 (0–3)
WPS, median score (IQR)	1 (0–2)	2 (1–4)	1 (0–2)	0 (0–2)	1 (0–2)
30-day mortality, n (%)	332 (5.6%)	121 (16.3%)	165 (4.3%)	14 (1.6%)	32 (7.2%)

### Albumin level

**Figure 1:**
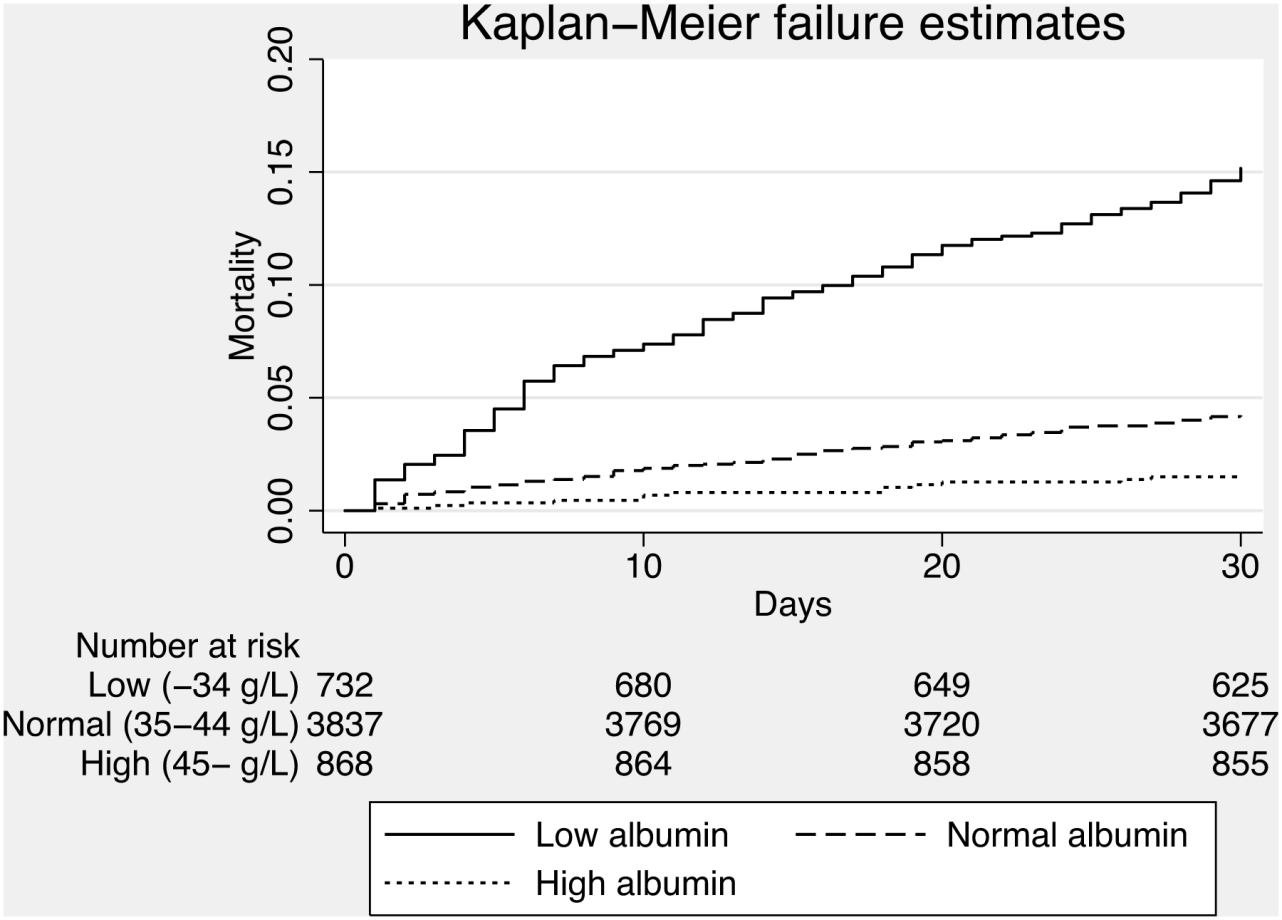
Kaplan-Meier failure plot of mortality of each of the three levels of albuminemia.

Albumin was analyzed in 5451 (92.5%). Median plasma albumin was 40 g/L (37–43). Patients with low albumin (<35 g/L) were significantly older than patients with normal albumin (p<0.001), had higher 30-day mortality (p<0.001) and were admitted longer (p<0.001), see table 1 and figure 1. Patients with high albumin (>44 g/L) were significantly younger (p<0.001) than patients with normal albumin, had a lower 30-day mortality and had a shorter hospital stay compared to than patients with normal albumin, see table 1 and figure 1.

### Summary measures

**Figure 2:**
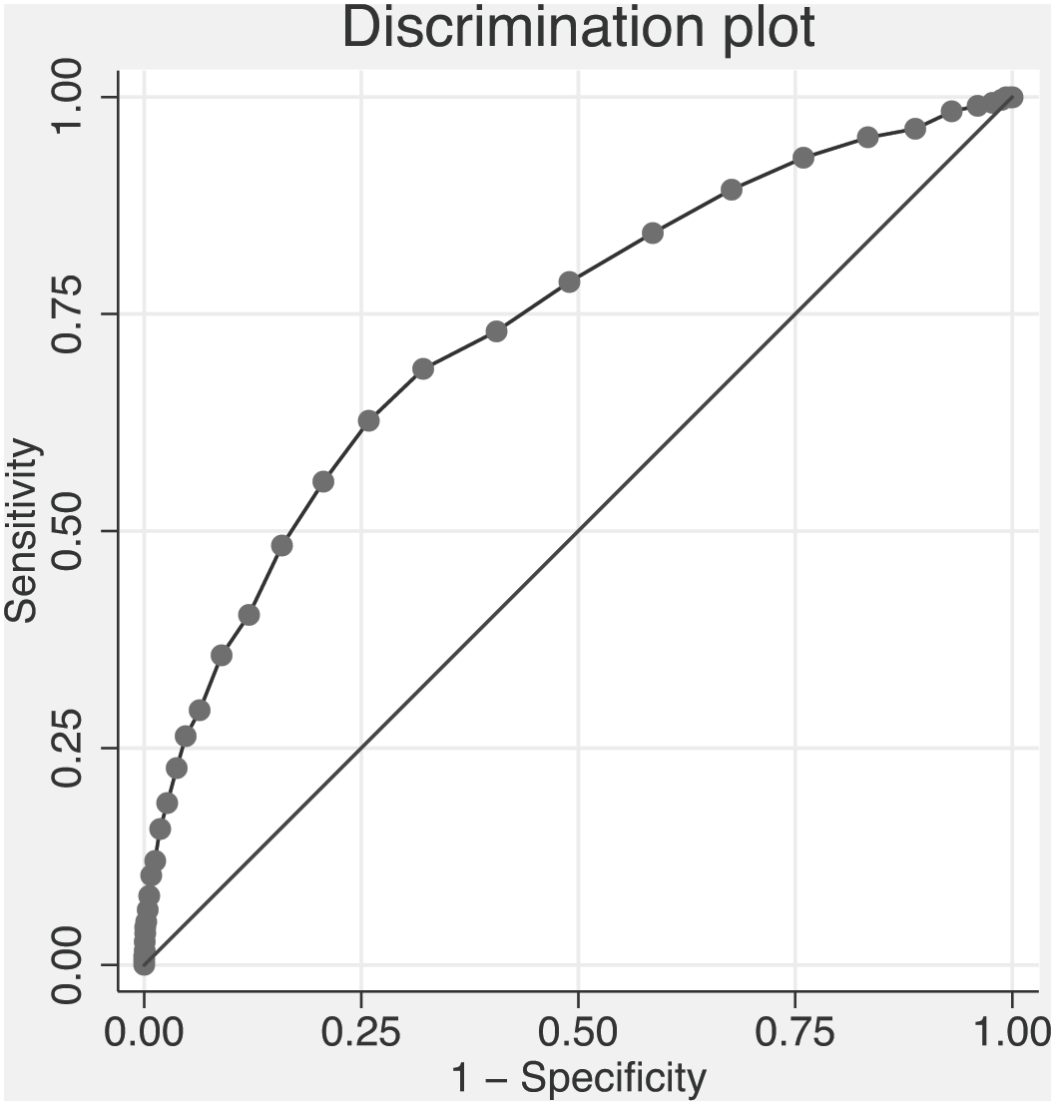
Discrimination plot of albumin as a predictor of 30-day all-cause mortality.

Discriminatory power was acceptable as AUROC was 0.73 (95% CI, 0.70–0.77), see figure 2. Calibration was good, Chi-square 6.85 (8 degrees of freedom), p = 0.55.

Hypoalbuminemia (<35 g/L) had sensitivity for 30-day mortality of 40.3% (95% Confidence Interval [CI], 34.7–46.1%), specificity of 87.9% (95% CI, 87.0–88.8%), positive predictive value of 16.3% (95% CI, 13.7–19.2%) and a negative predictive value of 96.2% (95% CI, 95.6–96.7%). Odds ratio for 30-day mortality with hypoalbuminemia was 4.34 (95% CI, 3.38–5.57).

### Multivariable logistic regression analyses

Hypoalbuminemia in it self had an odds ratio (OR) for 30-day all-cause mortality of 4.34 (95% CI, 3.38–5.57), see table 2. Controlling for age, sex, Charlson comorbidity score and WPS reduced the OR to 1.95 (95% CI, 1.31–2.90), see table 2.

**Table 2:** Logistic regression analyses of hypoalbuminemia with 30-day all-cause mortality as endpoint.

	Unadjusted Odds Ratio (95% Confidence Interval)	Adjusted^a^ Odd Ratio (95% Confidence Interval)
Normal albumin (35–44 g/L)	1.0 (reference)	1.0 (reference)
Low albumin (<35 g/L)	4.34 (3.38–5.57)	1.95 (1.31–2.90)
High albumin (>44 g/L)	0.36 (0.21–0.63)	0.58 (0.25–1.37)
Albumin as a continuous variable (g/L)	0.85 (0.83–0.86)	0.90 (0.87–0.93)

a sex, age, Charlson comorbidity score (continuous), Worthing Physiological Score (continuous).

### Acute and chronic inflammation

Adjusting the OR for hypoalbuminemia had very limited effect, see table 3. Overall, adjusting for CRP, liver disease, renal disease, cancer and rheumatologic disease only reduced the OR from 4.34 to 3.91, see table 3.

**Table 3:** Logistic regression of the effect of chronic or acute inflammation on hypoalbuminemia at admission.

Controlling for	Number of patients (%)	Odds ratio (95% Confidence interval) for hypoalbuminemia
Albumin alone	5,451 (100%)	4.34 (3.38–5.57)
Mild to moderate liver disease	231 (4.2%)	4.48 (3.49–5.76)
Severe liver disease	51 (0.9%)	4.34 (3.38–5.59)
Kidney disease	276 (5.1%)	4.34 (3.38–5.57)
Cancer with solid tumors	875 (16.1%)	3.98 (3.09–5.13)
Cancer with metastasis	156 (2.9%)	4.24 (3.30–5.45)
Rheumatologic disease	374 (6.7%)	4.32 (3.36–5.54)
C-reactive protein	5,542 (94.1%)	3.35 (2.56–4.38)

## Discussion

In this large cohort study of unselected acutely admitted medical patients, we found hypoalbuminemia to correlate strongly with 30-day mortality. Also, hypoalbuminemia had a good negative predictive power of 30-day all-cause mortality and did do so with acceptable precision. However, negative predictive power will automatically be high because of the number of events in the study. Thus, the emergency physician could use plasma albumin as a predictive marker when assessing patients upon arrival to the Emergency Department.

One of the key decisions made by physicians when patients arrive at a hospital is whether they should be admitted or not. However, this is not a simple task and many factors have to be taken into consideration. To assist in this process, several scoring systems using primarily vital signs and multiple blood tests have been developed. [9] Only a few of these prediction systems have been validated, and the clinical implications are unknown. However, in the initial development and validation cohorts, several of the systems showed a discriminatory power similar to hypoalbuminemia. This could mean that a low serum albumin upon arrival to the hospital is just a strong predictor of mortality as vital signs or a combination of other blood tests. Using existing prediction systems involves some level of calculation. Most systems require several parameters and most use some level of weighting. This limits the extent to which mental calculation may be carried out and thus the intuitiveness of use. However, perhaps we can get just as much information from a low serum albumin? If so, albumin should be included in the standard tests drawn on all medical patients upon arrival. Further, prospective, studies are needed to clarify this, but the thought is compelling.

We have not systematically assessed why hypoalbuminemia is such a strong predictor of 30-day mortality. Albumin is primarily a binding protein. Especially in the elderly, this is important, as the concentration of unbound drugs in the circulation is increased and the increased bioavailability may lead to adverse effects in hypoalbuminemia. [10] We found that patients with hypoalbuminemia were significantly older than patients with normal or increased albumin and had increased mortality. We did not have access to albumin measurements prior to admission. In an attempt to control for chronic hypoalbuminemia, we performed a series of analyses with the intent of adjusting for this. We added conditions identified under the Charlson comorbidity index as covariates with the possibility of chronic inflammation (e.g., liver, kidney and rheumatologic disease as well as cancer) as covariates to logistic regression analyses. This had very little effect on the OR, indicating a limited impact overall.

From previous studies, we know that hypoalbuminemia is associated with increased complications and worse prognosis in medical patients. [1], [2], [11], [12] We confirmed the prognostic implications of hypoalbuminemia on 30-day mortality.

Our study has several strengths. First, we present a large cohort of unselected medical patients. Second, we have complete follow-up due to the unique Danish registries. Third, we were able to include the majority of our patients as more than 90% had plasma albumin drawn. Alas, we also have some limitations. We were unable to report albumin prior to admission and thus estimate if any hypoalbuminemia was acute or chronic. Also, we are unable to report any development in plasma albumin during the admission. It would be interesting to assess other potential confounders in our regression analyses. However, we only had access to a limited number of these and more should be included in further studies. Last, this is a single center study from a Danish teaching hospital reporting only on medical patients. This reduces the generalizability of our findings.

## Conclusion

Hypoalbuminemia is associated with increased 30-day all-cause mortality in acutely admitted medical patients. Used as predictive tool for mortality, plasma albumin has acceptable discriminatory power and good calibration.
